# Molecular Characterization of a Novel *Staphylococcus Aureus* Surface Protein (SasC) Involved in Cell Aggregation and Biofilm Accumulation

**DOI:** 10.1371/journal.pone.0007567

**Published:** 2009-10-23

**Authors:** Katrin Schroeder, Mario Jularic, Samantha M. Horsburgh, Nina Hirschhausen, Claudia Neumann, Anne Bertling, Anja Schulte, Simon Foster, Beate E. Kehrel, Georg Peters, Christine Heilmann

**Affiliations:** 1 Institute of Medical Microbiology, University Hospital of Münster, Münster, Germany; 2 Department of Anaesthesiology and Intensive Care, Experimental and Clinical Haemostasis, University Hospital of Münster, Münster, Germany; 3 Department of Molecular Biology and Biotechnology, University of Sheffield, Sheffield, United Kingdom; Max Planck Institute for Infection Biology, Germany

## Abstract

**Background:**

Staphylococci belong to the most important pathogens causing implant-associated infections. Colonization of the implanted medical devices by the formation of a three-dimensional structure made of bacteria and host material called biofilm is considered the most critical factor in these infections. To form a biofilm, bacteria first attach to the surface of the medical device, and then proliferate and accumulate into multilayered cell clusters. Biofilm accumulation may be mediated by polysaccharide and protein factors.

**Methology/Principal Findings:**

The information on *Staphylococcus aureus* protein factors involved in biofilm accumulation is limited, therefore, we searched the *S. aureus* Col genome for LPXTG-motif containing potential surface proteins and chose the so far uncharacterized *S. aureus* surface protein C (SasC) for further investigation. The deduced SasC sequence consists of 2186 amino acids with a molecular mass of 238 kDa and has features typical of Gram-positive surface proteins, such as an N-terminal signal peptide, a C-terminal LPXTG cell wall anchorage motif, and a repeat region consisting of 17 repeats similar to the domain of unknown function 1542 (DUF1542). We heterologously expressed *sasC* in *Staphylococcus carnosus*, which led to the formation of huge cell aggregates indicative of intercellular adhesion and biofilm accumulation. To localize the domain conferring cell aggregation, we expressed two subclones of *sasC* encoding either the N-terminal domain including a motif that is found in various architectures (FIVAR) or 8 of the DUF1542 repeats. SasC or its N-terminal domain, but not the DUF1542 repeat region conferred production of huge cell aggregates, higher attachment to polystyrene, and enhanced biofilm formation to *S. carnosus* and *S. aureus*. SasC does not mediate binding to fibrinogen, thrombospondin-1, von Willebrand factor, or platelets as determined by flow cytometry.

**Conclusions/Significance:**

Thus, SasC represents a novel *S. aureus* protein factor involved in cell aggregation and biofilm formation, which may play an important role in colonization during infection with this important pathogen.

## Introduction

In the past two decades, *Staphylococcus aureus* has emerged one of the most important pathogens causing infections with indwelling medical devices, such as prosthetic joints, prosthetic heart valves, intravascular catheters, and cerebrospinal fluid shunts, which creates an increasing health care problem [1]. For example, prosthetic joint infections occur at a frequency of 1.5–2.5% in primary total hip or total knee arthroplasty with a mortality rate of up to 2.5% [2]. By far the most frequently isolated species from these infections are *Staphylococcus* species, i.e. *S. aureus* (22–39%) and coagulase-negative staphylococci (15–37.5%) [2].

The pathogenesis of device-associated infections with staphylococci is mainly characterized by the pathogens ability to colonize the surfaces of the implanted medical device by the formation of a three-dimensional structure of microorganisms embedded in a thick extracellular matrix composed of polysaccharides, proteins, extracellular DNA, and host factors, known as biofilm [3], [4], [5]. Microorganisms within a biofilm are protected against antimicrobial chemotherapy as well as against the immune system of the host.

Biofilm formation occurs in a two-step process. The first step involves the adherence of the bacteria to artificial surfaces that can occur either directly or via host factors acting as bridging molecules, such as the extracellular matrix and plasma proteins fibrinogen (Fg) and fibronectin (Fn) or platelets [6]. In the second step, the bacteria proliferate and accumulate into a biofilm requiring intercellular adhesion. Direct *S. aureus* adherence to the unmodified artificial surface may be mediated by the major autolysin Atl [7], which is highly homologous to the *S. epidermidis* autolysin/adhesin AtlE shown to be involved in the attachment to polymer surfaces [8]. *S. aureus* host-factor binding proteins that typically belong to the family of microbial surface components recognizing adhesive matrix molecules (MSCRAMM) are involved in binding to host factor-coated foreign material, among them Fn-binding proteins (FnBpA, FnBpB, Ebh), Fg-binding proteins (ClfA, ClfB), collagen-binding protein (Cna), bone-sialoprotein-binding protein (Bbp), and von Willebrand factor (vWf)-binding protein A (Spa) [9], [10], [11], [12], [13], [14], [15], [16]. Staphylococcal biofilm accumulation is mediated by polysaccharide as well as protein factors. The intercellular polysaccharide adhesin (PIA), a β-1,6-N-acetylglucosaminoglycan [3], is produced by the gene products encoded by the *icaADBC* operon that was first identified in *S. epidermidis* [17] and is also present in *S. aureus* [18]. Surface proteins conferring biofilm accumulation include the accumulation-associated protein (Aap) from *S. epidermidis* [19], [20] and the homologous *S. aureus* surface protein G (SasG) [21]. In *S. aureus*, another protein, the biofilm-associated protein (Bap), is involved in biofilm accumulation [22]. However, so far the *bap* gene has not been found in any *S. aureus* isolate of human origin, but has only been identified within bovine mastitis isolates [22].

A recent study demonstrated that all 18 *S. aureus* isolates from prosthetic joint infections carry the *icaADBC* operon, produce PIA, and are biofilm-positive [23]. Surprisingly, the biofilms of all 18 *S. aureus* isolates could be almost completely eradicated by the treatment with dispersin B (DspB), an enzyme with specific β-1,6-hexosaminidase activity as well as by the treatment with trypsin suggesting that both, proteinaceous adhesins and PIA contribute to biofilm formation in these *S. aureus* isolates. This was in contrast to *S. epidermidis* isolates from prosthetic joint infections. Only 62% of the 52 *S. epidermidis* isolates carry the *icaADBC* operon and *S. epidermidis* biofilms produced by *icaADBC*-positive strains were disintegrated by DspB, but not by proteases. Furthermore, biofilms produced by *icaADBC*-negative strains were disintegrated by proteases, but not by DspB [23]. Thus, different mechanisms seem to be involved in biofilm formation in clinical *S. aureus-* and *S. epidermidis-*associated prosthetic joint infection isolates. More specifically, in *S. aureus*-associated prosthetic joint infections, polysaccharide and protein factors seem to act synergistically in biofilm formation. Only 33% of the analyzed *S. aureus* strains carry the *sasG* gene and none of them carry the *bap* gene, indicating the existence of further, not yet identified surface proteins involved in biofilm accumulation of *S. aureus*.

Upon a search for LPXTG-motif containing surface-anchored proteins encoded by the *S. aureus* Col genome (http://www.tigr.org), we chose to study the so far uncharacterized SasC. We heterologously expressed *sasC* from *S. aureus* Col and from the clinical *S. aureus* isolate 4074 in *Staphylococcus carnosus* under the control of a xylose-inducible promoter. *S. carnosus* expressing *sasC* formed huge cell aggregates indicative of intercellular adhesion, which were disintegrated by protease treatment. Upon plasmid-encoded expression of *sasC*, *S. carnosus* as well as *S. aureus* not only formed huge cell aggregates, but also formed a much more pronounced biofilm in microtiter plates as well as in glass tubes than the respective wild-type strains. The domain conferring cell aggregation and biofilm formation was localized to the N-terminal domain of SasC. In conclusion, we identified SasC as a novel *S. aureus* factor involved in intercellular adhesion and biofilm accumulation.

## Results

### Identification and cloning of the *sasC* gene of *S. aureus*

As a candidate gene conferring biofilm formation in *S. aureus*, we amplified a DNA fragment containing the *sasC* gene including the ribosome binding site by polymerase chain reaction (PCR) from *S. aureus* 4074 and *S. aureus* Col genomic DNA using the primers CHsasCfor and CHsasCrev yielding 6577 bp DNA fragments. The DNA fragments were cloned into the *Bam*HI and *Sma*I sites of the vector pCX19 in *S. carnosus*, creating plasmids pSasC4074 or pSasCCol.

### Nucleotide sequence of *sasC* and amino acid sequence analysis of the deduced protein

The nucleotide sequence of the cloned *sasC* gene from *S. aureus* 4074 was determined on both strands. *sasC* consists of 6558 nucleotides and encodes a deduced protein of 2186 amino acids (aa) with a predicted molecular mass of 237.9 kDa. The ATG start codon is preceded by a putative ribosome binding site at a distance of 8 bp. Putative −10 (TATATT, nucleotides −61 to −56) and −35 (TAAACA, nucleotides −80 to −75) promotor sequences were deduced from homologous DNA sequences from strain *S. aureus* Col. A putative ρ-independent terminator consisting of two stem-loops is located downstream of the TAA stop codon. The deduced SasC sequence contains a putative signal peptide in the first 37 aa that contains an YSIRK motif, which seems to play a role in signal peptide processing [24]. The predicted *sasC* gene product is composed of 25.1% hydrophobic, 12.1% basic, and 13.2% acidic aa. The theoretical pI value of SasC is 5.08. The deduced aa sequence of SasC of strain 4074 shares 97% identical aa with homologous proteins from strains *S. aureus* MW2 [25] and MSSA476 [26], 96% identical aa with strains USA300 [27], COL [28], Newman [29], NCTC8325 (accession number: Q2FXH4 [30]), and N315 [31] and 89% identical aa with strain MRSA252 [26]. Besides, SasC shares 31% identical aa with Mrp and FmtB proteins from strain Col that have been implicated in methicillin-resistance [32], [33].

**Figure 1:**
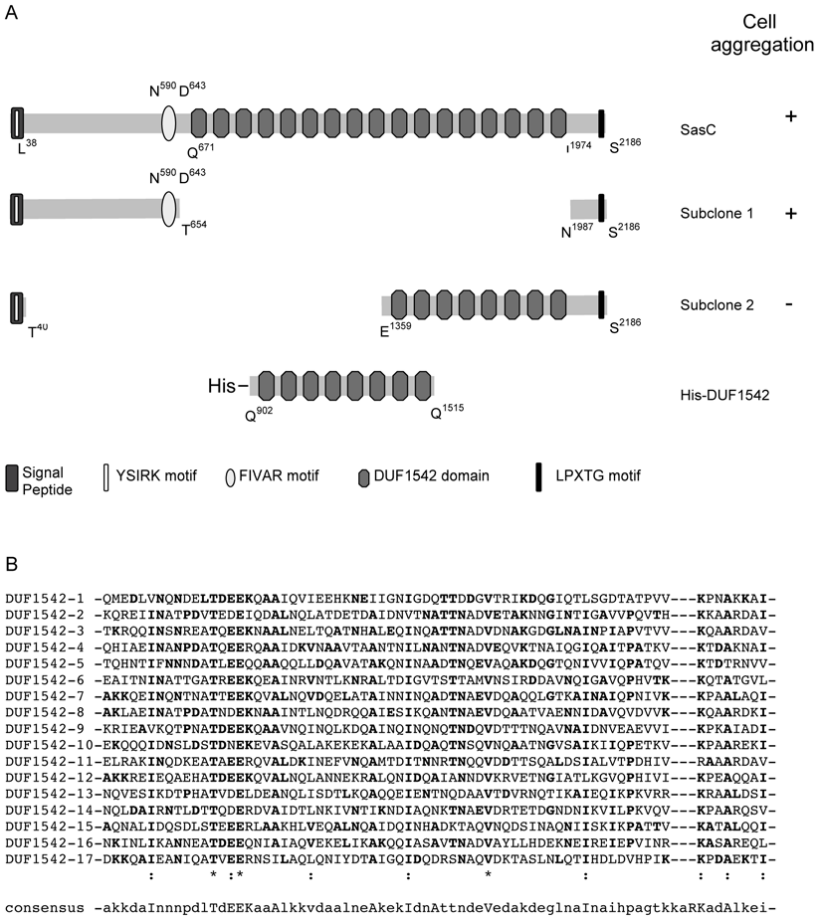
Schematic model of SasC and amino acid alignment of the DUF1542 repeats. A: Model of SasC. The positions of the N-terminal signal peptide including the YSIRK motif, the C-terminal LPXTG motif, the FIVAR motif (aa N-590 to D-643), and the 17 DUF1542 repeats (aa Q-671 to I-1974) are indicated. The DUF1542 repeats consist of 72 aa each separated by 5 aa. The SasC domains expressed by subclone 1 (aa T-654 is fused to N-1987) and subclone 2 (aa T-40 is fused to E-1359) are indicated. The SasC domain expressed as His-tagged fusion protein is shown: His-DUF1542 (aa Q-902 to Q-1515). + indicates cell aggregation mediated by the respective clone; − indicates no cell aggregation. B: Alignment of the deduced amino acid sequences of the DUF1542 repeats. The consensus sequence indicates the DUF1542 domain (Pfam accession number: PF07564). Bold letters indicate amino acids that match the consensus sequence. Asterics indicate identical amino acids, colons indicate very similar amino acids. Gaps (dashes) were filled in to maximize homologies.

Sequence comparison of the deduced SasC sequence with known protein sequences in databases revealed a domain structure of SasC (Fig. 1A). The central portion of SasC contains a domain, which is similar to the motif found in various architectures (FIVAR; 54 aa, starting at N-590 and ending at D-643). The FIVAR domain is followed by 17 direct repeated sequences of 72 aa each separated by a stretch of 5 aa, which are homologous to a domain of unknown function (DUF1542) also found in other cell surface proteins. The first DUF1542 repeat starts at Q-671 and the last ends at I-1974. The DUF1542 repeats share between 18 and 47% identical aa (Fig. 1B). The FIVAR motif and the DUF1542 domain are also present within Mrp and FmtB (see above) as well as within the cell surface protein Ebh from *S. aureus* [10] and the homologous Embp from *S. epidermidis* [34].

### SasC mediates strong cell aggregation in *S. carnosus*

**Figure 2:**
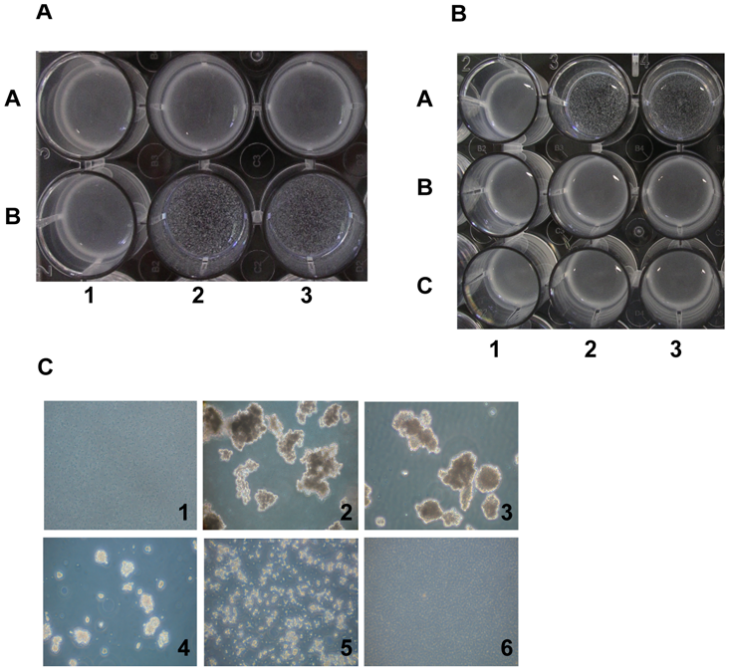
SasC-mediated cell aggregation of *S. carnosus*. Cell aggregation is visible macroscopically (A, B) and microscopically (C) and is protease-sensitive (B, C). A: Visible cell cluster formation in liquid medium. Strains were incubated overnight in TSB with (B) or without (A) 1% xylose with shaking. The cells were harvested by centrifugation, resuspended in PBS, and observed in the wells of 24-well microtiter plates. 1: *S. carnosus* (pCX19); 2, *S. carnosus* (pSasCCol); 3, *S. carnosus* (pSasC4074). B: After overnight growth in TSB with 1% xylose, *S. carnosus* cells were treated for 3 h at 37°C with PBS (A), 2.5 mg/ml trypsin (B), or 2 mg/ml proteinase K (C). 1: *S. carnosus* (pCX19); 2, *S. carnosus* (pSasCCol); 3, *S. carnosus* (pSasC4074). C: Phase-contrast micrographs. Cells were treated overnight at 37°C with different concentrations of trypsin (0.25 mg/ml, 3; 0.5 mg/ml, 4; 1.25 mg/ml, 5; 2.5 mg/ml, 6) or left untreated (1, 2). 1: *S. carnosus* (pCX19); 2–6: *S. carnosus* (pSasC4074).

After overnight growth in tryptic soy (TS) broth supplemented with 1% xylose, *S. carnosus* expressing *sasC* formed huge cell clusters that were visible macroscopically (Fig. 2A) and microscopically (Fig. 2C). In contrast, the strains did not form cell clusters without induction by xylose (Fig. 2A). The cell clusters were dissolved upon treatment with trypsin (Fig. 2B and C) or proteinase K (Fig. 2B). Disruption of cell clusters by trypsin was concentration-dependent (Fig. 2C).

### Characterization of *S. carnosus* and *S. aureus* expressing *sasC* or *sasC*-subclones

In order to dissect the functional domains within SasC, we constructed subclones of *sasC* expressing either the N-terminal domain including the FIVAR motif (subclone 1) or 8 of the 17 DUF1542 repeats (subclone 2) in *S. carnosus* yielding *S. carnosus* (pSasCsub1) or *S. carnosus* (pSasCsub2), respectively (see Fig. 1). An inverse PCR with the plasmid pSasC4074 as a template was carried out for the construction of subclone 1 and subclone 2 by using the primers CHsasCSub1rev/CHsasCSub1for and CHsasCSub2rev/CHsasCSub2for, respectively.

The resulting PCR fragments were ligated and subsequently, *S. carnosus* was transformed with the ligation mixtures. Correct clones were verified by PCR and DNA sequencing. However, DNA sequence analysis of subclone 1 revealed a 33-bp deletion in the C-terminal region so that aa T-654 is fused to N-1987 rather than to D-1976 (Fig. 1A). To functionally characterize *sasC* also in the *S. aureus* background, we transformed *S. aureus* 4074 and *S. aureus* SH1000, which is a 8325-4 derivative reconstituted for its *rsbU* mutation [35], with the plasmids pSasC4074, pSasCsub1, or pSasCsub2 leading to overexpression of *sasC* and its subfragments in these strains.

**Figure 3:**
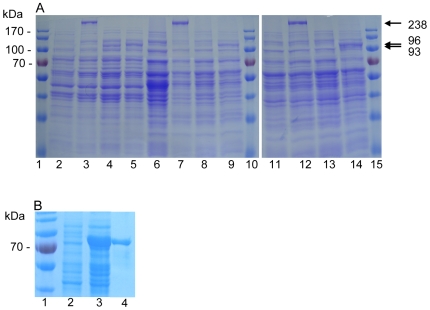
Expression of *sasC* or *sasC* subfragments. A: SDS-PAGE (10% separation gel) of cell lysates of *S. carnosus* or *S. aureus* expressing *sasC* or *sasC* subfragments. The lanes contained: 1, 10, 15, marker proteins; 2, *S. carnosus* (pCX19); 3, *S. carnosus* (pSasC4074); 4, *S. carnosus* (pSasCsub1); 5, *S. carnosus* (pSasCsub2); 6, *S. aureus* SH1000; 7, *S. aureus* SH1000 (pSasC4074); 8, *S. aureus* SH1000 (pSasCsub1); 9, *S. aureus* SH1000 (pSasCsub2); 11, *S. aureus* 4074; 12, *S. aureus* 4074 (pSasC4074); 13, *S. aureus* 4074 (pSasCsub1); 14, *S. aureus* 4074 (pSasCsub2). The sizes of marker proteins are shown on the left (prestained protein ladder; Fermentas, Leon-Rot, Germany); in the right margin the sizes (kilodaltons) of SasC and truncated SasC proteins are indicated. B: Expression and purification of 6 x His-DUF1542. SDS-PAGE (10% separation gel) of crude cell lysates from non-induced *E. coli* (pHis-DUF1542) (lane 2), IPTG-induced *E. coli* (pHis-DUF1542) (lane 3), and purified 6 x His-DUF1542 (1.5 µg) (lane 4). The size of one marker protein is shown on the left (lane 1; prestained protein ladder, Fermentas).

To verify the production of the whole SasC or the truncated SasC proteins, cell lysates of the strains were prepared and analyzed by SDS-PAGE (Fig. 3A). *S. carnosus* and the *S. aureus* strains expressing *sasC* revealed an additional protein band corresponding to the size of SasC of 238 kDa. The strains expressing subfragments 1 or 2 revealed additional protein bands at 93 or 96 kDa, respectively. The strains expressing *sasC* or the subfragments 1 or 2 were further characterized.

### (i) Cell aggregation

**Figure 4:**
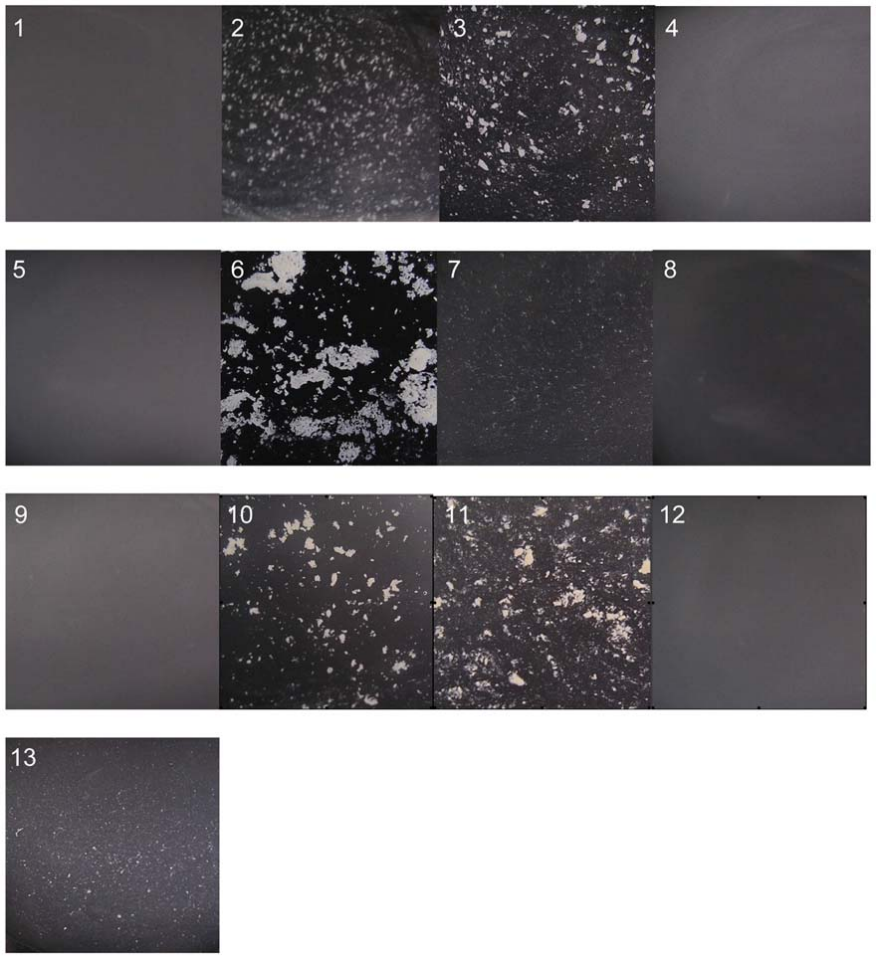
The N-terminal SasC domain, but not the DUF1542 repeats mediate cell aggregation. The strains were grown 24 h on TS agar plates containing 1% xylose and 10 µg/ml chloramphenicol, when appropriate. Then, a loop of cell material was resuspended in PBS buffer on a glass slide, viewed macroscopically and photographed against a dark background. 1, *S. carnosus* (pCX19); 2, *S. carnosus* (pSasC4074); 3, *S. carnosus* (pSasCsub1); 4, *S. carnosus* (pSasCsub2); 5, *S. aureus* 4074; 6, *S. aureus* 4074 (pSasC4074); 7, *S. aureus* 4074 (pSasCsub1); 8, *S. aureus* 4074 (pSasCsub2); 9, *S. aureus* SH1000; 10, *S. aureus* SH1000 (pSasC4074); 11, *S. aureus* SH1000 (pSasCsub1); 12, *S. aureus* SH1000 (pSasCsub2); 13, *S. carnosus* (pCN27). Large cell clusters are formed by strains expressing *sasC* and the subfragment 1, but not by strains expressing the subfragment 2.

In contrast to the wild-type strains, *S. carnosus* (pSasC4074), *S. aureus* 4074 (pSasC4074) and *S. aureus* SH1000 (pSasC4074) as well as *S. carnosus* (pSasCsub1) and *S. aureus* SH1000 (pSasCsub1) formed huge cell aggregates that were visible macroscopically in cultures grown overnight on TS agar supplemented with 1% xylose and resuspended in PBS on glass slides (Fig. 4). The cell aggregates formed by *S. aureus* 4074 (pSasCsub1) were somewhat smaller in size (comparable to those mediated by the *icaADBC* operon encoding the production of the polysaccharide intercellular adhesin, PIA [17] Fig. 4–13) corresponding to a lower expression level of the fusion protein (see Fig. 3). In contrast, the parent strains or strains expressing the subfragment 2 did not form visible cell aggregates (Fig. 4). Thus, *sasC* and its subfragment 1 confer cell aggregation.

**Figure 5:**
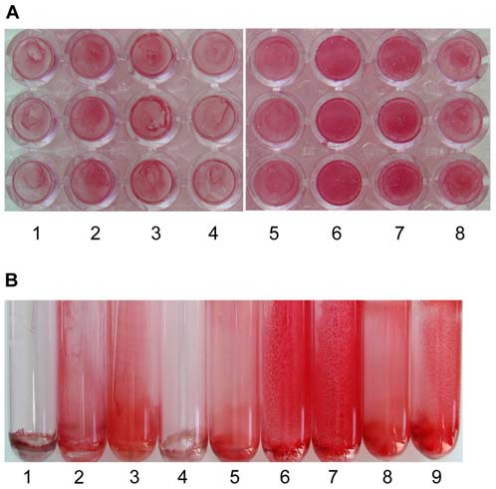
SasC-mediated biofilm formation of *S. carnosus* or *S. aureus* strains. A: Quantitative assay of biofilm formation. Lanes: 1, *S. carnosus* TM300 (pCX19); 2, *S. carnosus* (pSasC4074); 3, *S. carnosus* (pSasCSub1); 4, *S. carnosus* (pSasCSub2); 5, *S. aureus* SH1000; 6, *S. aureus* SH1000 (pSasC4074); 7, *S. aureus* SH1000 (pSasCSub1); 8, *S. aureus* SH1000 (pSasCSub2). B: Biofilm formation on glass. Lanes: 1, *S. carnosus* TM300 (pCX19); 2, *S. carnosus* (pSasC4074); 3, *S. carnosus* (pSasCSub1); 4, *S. carnosus* (pSasCSub2); 5, *S. aureus* SH1000; 6, *S. aureus* SH1000 (pSasC4074); 7, *S. aureus* SH1000 (pSasCSub1); 8, *S. aureus* SH1000 (pSasCSub2); 9, *S. carnosus* (pCN27).

**Figure 6:**
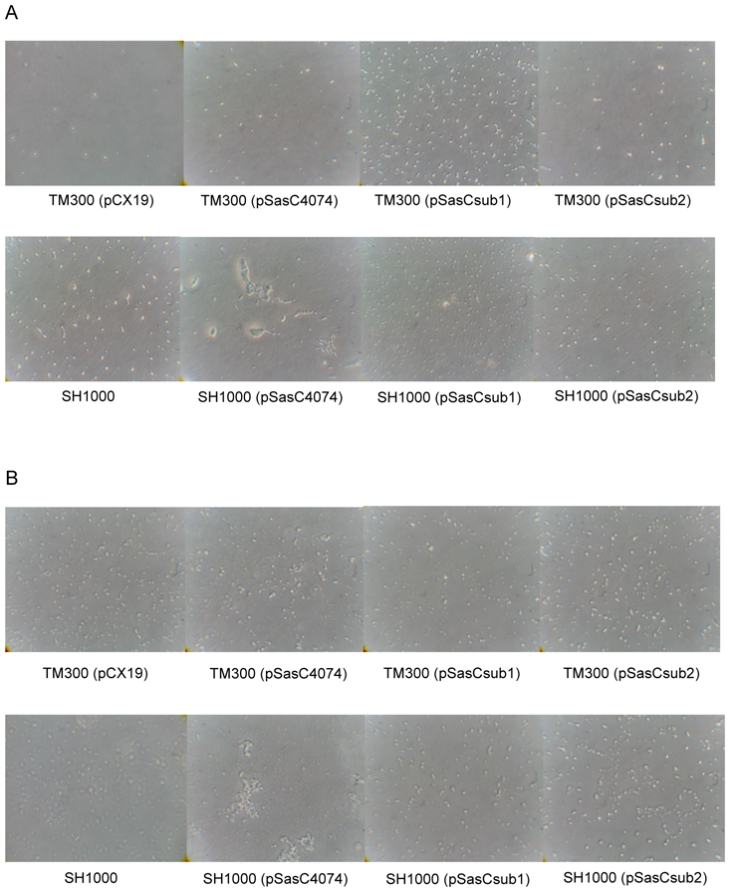
SasC-mediated initial attachment of *S. carnosus* or *S. aureus* strains. Phase-contrast micrographs of attached cells of *S. carnosus* (TM300) or *S. aureus* (SH1000) strains on polystyrene Petri dishes (A) or on glass slides (B).

**Figure 7:**
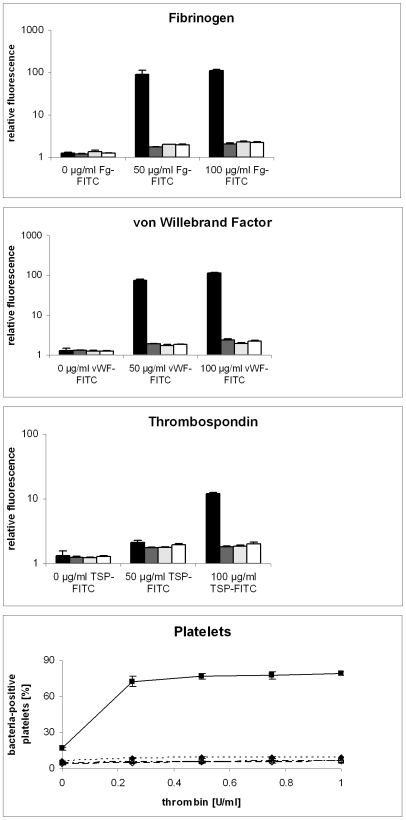
SasC does not bind to extracellular matrix proteins Fg, vWf, and TSP-1 or to platelets. Bars: black, *S. aureus* 4074; dark grey, *S. carnosus* (pCX19); light grey, *S. carnosus* (pSasC4074); white, *S. carnosus* (pSasCsub2). Solid black line: *S. aureus* 4074; small dashed line: *S. carnosus* (pCX19); dashed black line, *S. carnosus* (pSasC4074); dashed grey line, *S. carnosus* (pSasCsub2). The results represent the mean of three independent experiments. Standard deviations are indicated.

**Figure 8:**
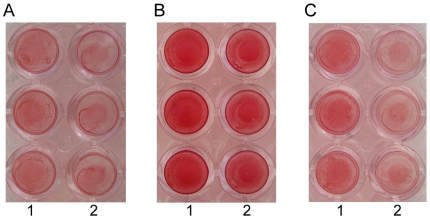
*S. aureus* SH1000 *sasC* shows reduced biofilm formation. Quantitative assay of biofilm formation. Lanes: 1, *S. aureus* SH1000; 2, *S. aureus* SH1000 *sasC*. A, 1% xylose; B, 0.25% glucose; C, no additional carbohydrate source.

**Figure 9:**
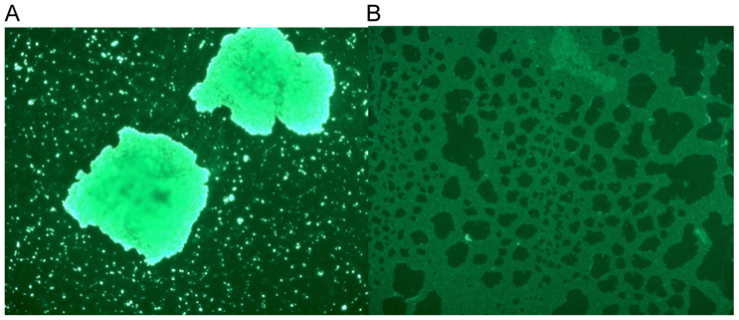
Detection of SasC by immunofluorescence microscopy. Strains grown in TSB were incubated with anti-His-DUF1542 antiserum raised in rabbits. Bound antibodies were detected with fluorescein-conjugated anti-rabbit F(ab′)_2_ fragment. Cells were viewed with a fluorescence microscope. *S. carnosus* (pSasC4074) cells reacted with the anti-His-DUF1542 antiserum, indicating the surface location of SasC. Magnification, ×400. A, *S. carnosus* (pSasC4074); B, *S. carnosus* (pCX19).

### (ii) Biofilm formation on polystyrene and glass

Biofilm formation on polystyrene was determined in the quantitative biofilm assay. Whilst *S. carnosus* (pCX19) revealed a biofilm-negative phenotype (*A*490 value: 0.11), *S. carnosus* (pSasC4074) and *S. carnosus* (pSasCSub1) showed an enhanced biofilm formation corresponding to an *A*490 value of 0.43 and 0.4 (Fig. 5A). In contrast, biofilm formation of strain *S. carnosus* (pSasCsub2) is comparable to that of the wild type (*A*490 value: 0.05).

In *S. aureus*, the expression of *sasC* or the subfragment 1 also led to a markedly increased biofilm formation as shown for strain SH1000 [*A*490 value of 1.5 or 1.4 for *S. aureus* SH1000 (pSasC4074) or *S. aureus* SH1000 (pSasCsub1), respectively, versus *A*490 value of 0.7 for *S. aureus* SH1000] (Fig. 5A). As observed with *S. carnosus*, the biofilm formation of *S. aureus* SH1000 (pSasCsub2) is comparable with that of its wild type (*A*490 value: 0.8). The results for *S. aureus* 4074 are similar (not shown).

The stronger capacity for biofilm formation mediated by *sasC* or the subfragment 1 also could be observed on a glass surface with *S. carnosus* as well as with *S. aureus* SH1000 (Fig. 5B) and *S. aureus* 4074 (not shown) and was comparable with that of *S. carnosus* (pCN27) producing PIA [17] (Fig. 5B). Thus, *sasC* and its subfragment 1 mediate biofilm formation on polystyrene and on glass.

### (iii) Initial attachment to polystyrene and to glass

Bacterial biofilm formation results from initial attachment of bacterial cells to a surface and subsequent accumulation into multilayered cell clusters, which requires intercellular adhesion visible as cell aggregation. To determine, whether *sasC* not only mediates cell aggregation, but also initial attachment, we analyzed the capacity of the respective strains for attachment to a polystyrene or a glass surface. Initial attachment of *S. carnosus* (pCX19) to polystyrene was very low indicated by a low number of attached bacteria (Fig. 6A). *S. carnosus* strains expressing *sasC* or its subfragments showed higher initial attachment with *S. carnosus* (pSasCsub1) yielding the highest numbers of attached bacteria (Fig. 6A). Essentially the same was observed with *S. aureus* SH1000 strains albeit at a higher level of attachment (Fig. 6A) and with *S. aureus* SH1000 (pSasCsub2) showing numbers of attached bacteria that were comparable with the wild type.

Initial attachment of the strains to a glass surface generally was higher (Fig. 6B) and similar with *S. carnosus* and *S. aureus* SH1000 wild-type strains and the same strains expressing *sasC* or the subfragment 2. In contrast, both strains expressing subfragment 1 showed a slightly lower number of attached bacterial cells (Fig. 6B). Thus, expression of *sasC* slightly increased attachment to a polystyrene surface, but did not increase attachment to glass.

### (iv) Binding to Fg, vWF, thrombospondin-1 (TSP-1), and platelets

The ability of *S. aureus* to bind to extracellular matrix and plasma proteins and to host cells determines its capacity for tissue colonization. The potential of *sasC* to mediate binding to the extracellular matrix and plasma proteins Fg, TSP-1, and vWf as well as to platelets was analyzed by flow cytometry. While *S. aureus* 4074 bound to Fg, TSP-1, and vWf as well as to activated platelets in a dose-dependent fashion, *S. carnosus, S. carnosus* (pSasC4074), and *S. carnosus* (pSasCsub2) did not (Fig. 7). Thus, *sasC* does not mediate binding to these extracellular matrix proteins or to activated platelets.

### Biofilm formation of a *sasC* transposon (Tn)*917* insertion mutant (SMH2035)

To further support the role for SasC in biofilm formation, we analyzed the capacity of a *sasC* Tn*917* insertion mutant (SMH2035) for biofilm formation in comparison to its wild-type strain *S. aureus* SH1000. Under all conditions tested, the *sasC* mutant strain showed reduced biofilm formation in microtiter plates, i.e. the addition of 1% xylose [*A*490 value of 0,4 for SMH2035 versus *A*490 value of 0,7 for SH1000], 0.25% glucose [*A*490 value of 1,6 for SMH2035 versus *A*490 value of 1,8 for SH1000] or no additional carbohydrate source in the biofilm assay [*A*490 value of 0,5 for SMH2035 versus *A*490 value of 1,0 for SH1000] (Fig. 8). The biofilm-forming capacity of both strains was most pronounced upon addition of 0.25% glucose, which is known to induce the production of PIA in *S. epidermidis* [36] and probably leads to enhanced PIA production also in *S. aureus*, which might partially obscure the function of SasC. The presence of 1% xylose seems to slightly reduce biofilm formation. So far, the reason for this is unknown.

### Expression and purification of the 6 x Histidine (His)-DUF1542 fusion protein in *Escherichia coli*

For expression of the *sasC* portion encoding 8 of the 17 DUF1542-repeats in *E. coli*, the PCR-amplified fragments were cloned into the expression vector pQE30Xa. One representative clone expressing the DUF1542 repeats (6 x His-DUF1542) contained the plasmid pHis-DUF1542. Subsequently, the 6 x His-DUF1542 fusion protein was purified from *E. coli* (pHis-DUF1542) via its His-tag using Ni-NTA affinity chromatography under native conditions. SDS-PAGE of the affinity-purified fusion proteins revealed an approximately 70-kDa protein for the 6 x His-DUF1542 fusion protein (Fig. 3B). A protein with the same size was present in cell lysates of an induced culture of *E. coli* (pHis-DUF1542) and absent from cell lysates of an induced culture of *E. coli* (pQE30) (not shown) and of a non-induced culture of *E. coli* (pHis-DUF1542) (Fig. 3B).

### Surface location of SasC

To detect the surface location of SasC, the antiserum against the 6 x His-DUF1542 fusion protein that was raised in rabbits was used in immunofluorescence microscopy. The anti-His-DUF1542 antiserum strongly reacted with cells of *S. carnosus* (pSasC4074) (Fig. 9) and *S. aureus* 4074 (pSasC4074) (not shown) indicating the cell surface location of SasC. With *S. carnosus* (pSasC4074), no immunofluorescence was detected with the preimmune serum (not shown) and with *S. carnosus* (pCX19), no immunofluorescence was detected with the anti-His-DUF1542 antiserum (Fig. 9). However, there was some immunofluorescence detectable with the preimmune serum and strain *S. aureus* 4074 (not shown), which may be due to the IgG-binding and surface-associated proteins A and Eap [37], [38].

### Characterization of *sasC* expression in *S. aureus* strains

To analyze the production of SasC in *S. aureus*, we performed Western blot analysis using the anti-His-DUF1542 antiserum. In cultures of *S. aureus* 4074, Col, and SH1000, a faint band corresponding to SasC was detected in lysostaphin lysates of cultures after 10, 16, 24, and 48 h of growth (not shown). A strong production of SasC was observed in control strains *S. aureus* 4074 (pSasC4074) and *S. carnosus* (pSasC4074), while no protein isolated from *S. carnosus* (pCX19) reacted with the anti-His-DUF1542 antiserum (not shown). SasC did not react with the preimmune serum (not shown).

### Prevalence of *sasC*

To determine the prevalence of the *sasC* gene among clinical *S. aureus* isolates, we performed PCR analysis using primers CHsasC1for and CHsasC1rev. An approximate 500 bp fragment encoding a portion of the N-terminal SasC domain was found to be present in 97% (66/68) of the clinical *S. aureus* strains that were tested (not shown). This indicates a very high prevalence of the *sasC* gene among clinical *S. aureus* isolates.

## Discussion

The most frequently isolated bacteria from implant-associated infections are *S. aureus* and coagulase-negative staphylococci causing significant morbidity and mortality. The pathogenicity of these infections is characterized by the pathogens pronounced ability to form biofilms. To date, several *S. aureus* genes have been implicated in biofilm formation, among them *atl* [7], *dltA* [39], and the *icaADBC* gene cluster [18]. *S. aureus* surface proteins reported to be involved in biofilm formation include SasG [21], the Fn- and Fg-binding proteins FnBPA and FnBPB [40], and the biofilm-associated protein Bap [22]. SasG is homologous to the accumulation-associated protein Aap, which mediates biofilm accumulation in *S. epidermidis* [19], [20], and the plasmin-sensitive surface protein Pls [41], which so far has not been implicated in biofilm formation. Furthermore, extracellular genomic DNA (eDNA) has been established as another important component of *S. aureus* biofilms [5]. eDNA is released from the bacteria by cell lysis and may implicate an additional role for the major *S. aureus* autolysin Atl in biofilm development besides its function in initial attachment [5].

However, a recent study indicated the existence of further, yet unidentified surface proteins contributing to *S. aureus* biofilm formation [23]. As a potential candidate, we identified SasC in the *S. aureus* genome and expressed its gene heterologously in *S. carnosus* as well as in the *S. aureus* strains SH1000 and 4074 under a xylose-inducible promotor. All strains expressing *sasC* showed a high-level production of a protein with a molecular size of approximately 240 kDa corresponding to SasC. Expression of *sasC* led to strong cell cluster formation, intercellular adhesion, and biofilm formation. Furthermore, a *sasC* Tn*917* insertion mutant (SMH2035) showed a reduced capability for biofilm formation in comparison to its wild type.

SasG and Aap promote biofilm formation via their B-repeats. Each B-repeat also known as G5 domain consists of 128 aa and is present 7 and 5 times in SasG and Aap, respectively [20], [21]. Recently, the G5 domains were found to be zinc-dependent adhesion modules and a “zinc zipper” mechanism was suggested for G5 domain-based intercellular adhesion in SasG- or Aap-mediated biofilm accumulation [42]. SasC also contains a repeat region with 17 repeats of 72 aa being similar to the DUF1542 domain (see Fig. 1). The SasC repeats do not share sequence similarities with the B-repeats of SasG and Aap and are not involved in biofilm formation. Instead in SasC, the domain conferring cell aggregation and biofilm formation could be localized to the N-terminal domain by subcloning experiments (see Fig. 1).

Furthermore, SasG and Aap must undergo proteolytic cleavage to become active, while SasC-mediated biofilm formation does not depend on proteolytic cleavage: the formation of SasC-mediated biofilms was unchanged in the presence of the protease inhibitor α2-macroglobulin (data not shown) that blocked SasG and Aap-mediated biofilm formation [20], [21]. Moreover, *S. carnosus* expressing *aap* only formed a detectable biofilm after treatment with 2 µg/ml of trypsin leading to the production of a truncated version of Aap or when an N-terminally truncated version of *aap* was cloned in *S. carnosus* [20]. In contrast, no truncation of SasC was involved in SasC-mediated biofilm formation. Thus, the mechanism of SasC-mediated biofilm accumulation clearly differs from that mediated by SasG/Aap. Because of the lack of sequence similarities, the SasC-mediated mechanism probably is also distinct from that mediated by Bap [22].

Recently, a role for FnBPA and FnBPB in biofilm accumulation, which is independent of their Fn- and Fg-binding activities was reported. Like with SasC, the domain conferring biofilm accumulation is located within the N-terminal domain (A domain), which also includes the domain for Fg-binding. However, biofilm formation seems to be independent of Fg-binding, because a single aa exchange within that region abolished Fg-binding, but did not influence the capability for biofilm formation [40]. Because the FnBPs and SasC do not share significant sequence similarities within their N-terminal domains, the mechanism of biofilm accumulation between the two also seems to differ.

The N-terminal SasC domain shows significant homology to the N-terminal domains of Mrp and FmtB, both of which have been reported to be involved in methicillin resistance [32], [33]. However, the mechanism of Mrp and FmtB conferring methicillin resistance is not completely clear yet and may be indirect. A function of these proteins in biofilm formation has not been proposed so far.

The mechanism of SasC-mediated biofilm accumulation is not known yet and may involve either protein-protein interactions conferred by the N-terminal SasC domain or protein-carbohydrate interactions conferred by the FIVAR motif. The FIVAR motif is located at the C-terminus of the N-terminal domain (see Fig. 1) and has been proposed to have a sugar-binding function. Further analyses are necessary to elucidate these possibilities.

SasC not only mediates cell cluster formation and intercellular adhesion, but also slightly increases the attachment of the cells to polystyrene. Initial attachment to a polystyrene surface depends on cell surface hydrophobicity [43], [44]. With 25.1% hydrophobic aa, the percentage of hydrophobic aa of SasC is comparable to that of AtlE (26.4%) previously found to mediate initial attachment to polystyrene in *S. epidermidis* [8]. Remarkably, the higher level of initial attachment to polystyrene observed with *S. carnosus* and *S. aureus* producing subclone 1 correlated with a higher percentage of hydrophobic aa (28.1%) and a lower percentage of charged aa (23.7%: 12.3% basic and 11.4% acidic aa) of subclone 1 in comparison to subclone 2, which contains 26.6% hydrophobic aa and 30.1% charged aa (14.7% basic and 15.4% acidic aa).

Although the *S. aureus* wild-type strains Col, SH1000, and 4074 all harbour the *sasC* gene, we were not able to detect SasC in lysostaphin lysates by SDS-PAGE, but only by Western immunoblot analysis indicating very low *sasC* expression *in vitro*. However, the identification of SasC as an *in vivo*-expressed antigen during infection delineates its potential clinical significance [45]. Thus, *sasC* expression may be induced *in vivo*. Several genes are involved in the regulation of *S. aureus* biofilm formation, such as *agr* [46], [47], *sarA* [48], [49], *sigB* [50], *rbf* [51], *tcaR* [52], *arlRS* [53], and *alsSD* [54]. Further experiments are necessary to characterize the expression of *sasC*.

Homologous *sasC* genes were found in the eight sequenced *S. aureus* genomes analyzed and the sequence similarities of the SasC gene products ranged from 89% to 97%. Consistently, we found a very high prevalence of the *sasC* gene among clinical *S. aureus* strains delineating the potential importance of SasC in colonization and infection. In conclusion, we identified and characterized a novel *S. aureus* surface protein, SasC, involved in cell aggregation and biofilm formation, which may play an important role in colonization during infection with this important pathogen.

## Materials and Methods

### Ethics Statement

This study was conducted according to the principles expressed in the Declaration of Helsinki. The study was approved by the local ethics committee (Ethikkommission der Aertzekammer Westfalen-Lippe und der Medizinische Fakultaet der WWU Muenster) (reference number: Sitzung 19.05.1999). All volunteers provided written informed consent for the collection of samples and subsequent analysis.

### Bacterial strains, plasmids, and media

The *sasC* gene was cloned from the clinical strain *S. aureus* 4074 [55] and from *S. aureus* Col [28]. *S. carnosus* TM300 [56], *S. aureus* 4074 [55], and *S. aureus* SH1000 [35] were used as cloning hosts. The *sasC* Tn*917* insertion mutant SMH2035 was isolated by sequencing Tn insertion junctions from a Tn*917* mutant library of *S. aureus* SH1000, using direct sequencing from isolated genomic DNA [57]. One of the mutants gave a junction sequence, which mapped to an insertion point at 1812698 in the *S. aureus* N315 genome. This corresponded to inactivation of SasC at aa 934. *E. coli* TG1 was used to construct the plasmid for the production of the 6 x His-DUF1542 fusion protein and its purification.

For the expression of *sasC* in staphylococci, the vector pCX19, a derivative of the xylose-inducible expression vector pCX15 [58] and for the production and purification of the 6 x His-DUF1542 fusion protein, the Qia*express* vector pQE30Xa (Qiagen) was used.

To determine the prevalence of *sasC*, a 489 bp internal *sasC* fragment was amplified from genomic DNA of 68 clinical *S. aureus* isolates obtained from patients at the University Hospital of Münster (Münster, Germany).

The staphylococci were routinely cultivated in TS broth (TSB). *E. coli* strains were grown in Luria-Bertani (LB) medium. TS and LB agar contained 1.4% agar. TSB and TS agar were supplemented with 1% xylose to induce *sasC* expression. Selection for resistance to antibiotics in *E. coli* was performed with 100 µg/ml ampicillin and in staphylococci with 10 µg/ml chloramphenicol or 5 µg/ml erythromycin, when appropriate. Wild-type strains not harboring a plasmid were grown in the presence of 0.07% ethanol in the respective assays, when compared to the strains harboring a plasmid to rule out an effect of the ethanol. Because the *sasC* Tn*917* insertion mutant SMH2035 is stable, no antibiotic or ethanol was included in the assays comparing the SH1000 strain and its *sasC* mutant.

Bovine serum albumin (BSA) and α-thrombin (bovine) were purchased from Sigma. Human Fg was purchased from Enzyme Research Labs. The monoclonal antibody against human CD42a (GP IX) (conjugated with phycoerythrin [PE]) was delivered by Exalpha via NatuTec. Syto 13 for labeling of staphylococcal cells was purchased from Molecular Probes via Mobitec.

### DNA manipulations, transformation, PCR, DNA sequencing, websites, and Pfam accession numbers

DNA manipulations and transformation of *E. coli* were performed according to standard procedures [59]. *S. carnosus* and *S. aureus* strains were transformed with plasmid DNA by protoplast transformation [60]. Plasmid DNA was isolated using the Qiagen Plasmid Kit and chromosomal DNA was isolated using the QIAamp DNA Blood Mini Kit according to the instructions of the manufacturer (Qiagen). PCR was carried out with the PCR Supermix High Fidelity (Invitrogen) or with the Expand Long Template PCR System (Roche, Mannheim, Germany) in accordance with the protocol of the supplier. The primers (see Table 1) were synthesized by MWG-Biotech (Ebersberg, Germany).

**Table 1:** Oligonucleotide primers used in this study.

Primer name	Oligonucleotide sequence (5′→3′)	
CHsasCfor	GTG AGA TCT CCA GGA GGA AAA CGA AAT GAA TTT G (*Bgl*II)	cloning *sasC*
CHsasCrev	CGT GGG CCC AAT TAT GAT TCT TTT TCG TTT TTA GTA CG C (*Sma*I)	cloning *sasC*
CHsasCDUFfor	CAA CAT ATC GCA GAG ATC AAT G	expression
CHsasCDUFrev	GTG GGA TCC TTA TTG TTG CTT AAC TGC ATC TCT AGC (*Bam*HI)	expression
CHsasCSub1rev	CGT ATG TTG CAT TTG ATT AG	subcloning *sasC*
CHsasCSub1for	GAT GAT CTT GCA CGC GTC AC	subcloning *sasC*
CHsasCSub2rev	CGT AGT TAA GGC TTG TGC ACC	subcloning *sasC*
CHsasCSub2for	GAA AAA GCT GTT AAA GAA AAG	subcloning *sasC*
CHsasC1for	GCA ACG AAT CAA GCA TTG G	prevalence
CHsasC1rev	TGA CAG CAC TTC GTT AGG	prevalence
pCX19for	CTA AAT CGA TTT CTG GCC C	sequencing
SasC60for	GTATTAGGAAGTATAAAGTAGG	sequencing
SasC700for	CCA ACA ACT GAT CCT AAT GCC	sequencing
Sub1SasC4620rev	CAT CAG TCG CAT GTT CCG C	sequencing
Sub2SasC1380for	GAATAATGGTAATTCTGGTG	sequencing
SasC840rev	CCATTGATTAGTGTGAAGCCC	sequencing
SasC2100rev	CCT CGA TAA CTT GTA TTG CTG C	sequencing
SasC3860for	GGC GAA GCG TAT TGA AGC GG	sequencing
SasC5040for	GAT GCA ATC CGA AAT ACG TTG G	sequencing

The DNA sequence of both strands of the *sasC* gene of the clinical isolate *S. aureus* 4074 was determined by MWG-Biotech using a LI-COR DNA sequencer.

The DNA and deduced protein sequences were analyzed using the program “JustBio” at http://www.justbio.com. The protein sequences were compared with those of known proteins using the programs BLASTP [61] and FASTA [62]. The alignments were done using the program ClustalW at the European Bioinformatics Institute (EBI, Cambridge, UK). The Pfam accession numbers are: PF04650 for the YSIRK_signal; PF07554 for the FIVAR motif; PF07564 for the DUF1542 domain; PF00746 for the Gram_pos_anchor (LPXTG_anchor) available at: http://pfam.sanger.ac.uk/ [63]. The signal peptide of SasC was predicted by using the program “SignalP” at http://www.cbs.dtu.dk/services/SignalP/).

### Biofilm formation assay

For quantification of the biofilm-forming capacity, a test for biofilm production was performed essentially as described previously [44]. Briefly, strains were grown in TSB supplemented with 1% xylose for 24 h at 37°C in 96-well polystyrene microtiter plates (cell star; Greiner, Frickenhausen, Germany), the wells were washed with phosphate-buffered saline (PBS) and adherent biofilms were stained with 0.1% safranin (Serva). In some experiments, instead of 1% xylose, 0.25% glucose or no additional carbohydrate source was added. Absorbance was measured with a Micro-ELISA-Autoreader at 492 nm. Strains were tested at least in triplicate. Determination of biofilm formation on a glass surface was carried out essentially in the same way, except that glass tubes were used instead of microtiter plates and 5 ml of TSB were inoculated instead of 200 µl.

### Initial adherence to a polystyrene or a glass surface

Initial cell attachment was tested as described previously [44]. Briefly, diluted cell suspensions of bacteria in PBS were incubated for 30 min in polystyrene Petri dishes (Sarstedt) at 37°C or on glass slides and after a washing procedure, attached bacteria were evaluated by phase-contrast microscopy.

### Construction and purification of the 6 x His-DUF1542 fusion protein and anti-His-DUF1542 antiserum

For the construction of the His-tagged fusion protein, the primers CHsasCDUFfor and CHsasCDUFrev were used to amplify 8 of the DUF1542-repeats of *sasC* from genomic DNA of *S. aureus* Col, introducing a *Bam*HI-site at the 3′ end. The PCR-amplified fragment was cloned into the vector pQE30Xa, so that the gene fragment is in frame with the His-codons. One representative clone expressing the DUF1542-repeats was designated *E. coli* (pHis-DUF1542) (see Fig. 1). The 6 x His-DUF1542 fusion protein was purified under native conditions via its His-tag using Ni-NTA affinity chromatography (Ni-NTA Spin Kit; Qiagen) according to the protocol of the suppliers (see Fig. 3B). The yield of the 6 x His-DUF1542 fusion protein was in the range of 150 µg per 10 ml culture volume as determined by the Coomassie Plus™ Protein Assay (PIERCE, Rockford, IL, USA distributed by Perbio Science, Bonn, Germany). The purified 6 x His-DUF1542 fusion protein was used to immunize rabbits by Eurogentec (Belgium) according to their standard immunization program.

### Protein isolation, SDS-PAGE, and Western blot analysis

Staphylococcal surface proteins covalently linked to the peptidoglycan were prepared from cultures that were grown overnight in TSB broth at 37°C by lysostaphin treatment. For this, the staphylococcal cells were harvested by centrifugation, washed and then, the cell pellet was resuspended in 20 ml Tris-buffered saline (TBS) pH 7,4 per g bacteria. After adding 500 µg lysostaphin (Ambi Products LLC, Lawrence, NY, USA; distributed by WAK Chemie, Steinbach, Germany) and 100 µg DNAse (Sigma) per g bacteria, the suspension was incubated at 37°C with shaking. Afterwards, the cell debris was removed by centrifugation (45 min, 13.000 rpm, 4°C). Lysostaphin lysates were stored at −20°C. Crude cell lysates of *E. coli* (pHis-DUF1542) were prepared from non-induced and induced (addition of 1 mM Isopropyl-β-D-thiogalactoside [IPTG] and continued growth of 4 h) cultures by harvesting the cells, resuspending the cell pellet in sample buffer, and heating the suspension for 5 min at 95°C. Additionally, as a negative control, a crude cell lysate was prepared from an induced culture of *E. coli* (pQE30). After centrifugation, 7 µl of the cell lysates from *E. coli*, 9 µl of the staphylococcal cell lysates, or 2 µl of the purified protein (containing 1.5 µg) were subjected to SDS-PAGE (10% separation gel and 4.5% stacking gel). Proteins were stained with Coomassie brilliant blue R250 (0.1%).

For Western immunoblot analysis, staphylococcal surface proteins, crude cell lysates, or purified proteins were prepared and separated by SDS-PAGE as described above and transferred to a nitrocellulose membrane (Schleicher and Schuell, Dassel, Germany). The membranes were then blocked in Tris-buffered saline (TBS)/3% BSA (overnight) and washed three times with TBS/0.5% Tween 20 (TBST). Afterwards, the nitrocellulose membranes were incubated for 2 h with the anti-His-DUF1542 antiserum diluted 1∶2000 in TBST/3% BSA. As a negative control, incubation was performed in TBST/3% BSA with (1∶2000) or without preimmune serum. The reaction of proteins with specific antibodies was detected by incubation (1 h) with anti-rabbit immunoglobulin G (IgG)/alkaline phosphatase (AP) conjugate (Dako GmbH, Hamburg, Germany) diluted 1∶5000 in TBST/3% BSA and subsequent color reaction.

### Purification and fluorescence-labelling of Fg, thrombospondin-1 (TSP-1), or von Willebrand factor (vWF)

Labelling of Fg was performed as described previously [64]. TSP-1 in an adhesive conformation was purified from freshly isolated human platelets as described before [65]. For labelling TSP-1 with fluorescein-isothiocyanate (FITC) (Calbiochem; La Jolla, CA, USA), FITC was added to TBS/2 mM CaCl_2_ containing TSP-1 in a molar ratio of 600∶1 and incubated for 24 h at 4°C. Unbound label was removed using a Sephadex G-25 PD-10 column equilibrated with TBS. The concentration of FITC-labeled TSP-1 was determined by using the Pierce BCA protein assay (Pierce Europe B.V., BA oud-Beijerland, The Netherlands) according to the manufacturers instructions. vWf was purified and labeled according to Hartleib *et al* [66].

### Flow cytometric analysis of Fg-FITC, TSP-1-FITC, or vWF-FITC binding to staphylococci

Measurement of the binding of Fg-FITC, TSP-1-FITC, or vWf-FITC to staphylococcal cells was analyzed as described before for the binding of vWf-FITC to *S. aureus* [66]. Briefly, bacteria from an overnight culture (120,000 cells/µl) were incubated with Fg-FITC, TSP-1-FITC, or vWf-FITC (final concentrations 0, 50, or 100 µg/ml) in TBS/2 mM CaCl_2_ for 10 min at room temperature. After washing and sonication, bacteria (5,000 cells/determination) were analyzed in a flow cytometer (Becton Dickinson, FACSCalibur flow cytometer, Heidelberg, Germany) using an excitation wave length of 488 nm at the FACSCalibur standard configuration with a 530 nm bandpass filter. Data were obtained from fluorescence channels in a logarithmic mode.

### Preparation of platelets

Blood was taken from healthy adult volunteers who had not taken any medication affecting platelet function for at least 2 weeks before the study. Platelet-rich plasma (PRP) was prepared from anticoagulated blood by centrifugation and the platelets were gel-filtered on a Sephadex Cl-2B column [67]. To inhibit fibrin polymerisation, experiments were performed in the presence of the peptide GPRP (1.25 mM) as described previously [67]. The platelets were labeled by incubation with a monoclonal anti-CD42a (GP IX) antibody conjugated with PE at saturated concentrations for 30 min.

### Preparation of bacteria and flow cytometric measurement of *Staphylococcus*-platelet associate formation

*Staphylococcus*-platelet associate formation was measured essentially as described before [68]. Briefly, bacteria grown over night were washed in TBS, briefly sonicated, and diluted with TBS/2 mM CaCl_2_ to 250,000 bacteria/µl. Bacteria were labeled with the fluorescent dye Syto 13 (emission similar to FITC) at a concentration of 2 µM for 10 min, washed in TBS and briefly sonicated again.

Platelets were activated with α-thrombin at the given concentrations for 4 min and subsequently, labeled bacteria were added. Bacteria and platelets (10∶1) were coincubated for 15 min at room temperature and conjugate formation was measured immediately thereafter in a flow cytometer. Associates were identified by double labelling with Syto 13 (FL-1, “FITC like” signal) and anti CD42a-PE (FL-2, PE signal), and given as the rate of bacteria-positive platelets. Given are the mean values of three independent experiments.

### Immunofluorescence microscopy

The detection of SasC by immunofluorescence microscopy was performed essentially as described before [17]. Briefly, cultures were grown aerobically in TSB for 16 h at 37°C. After washing, aliquots (30 µl) were applied to glass slides. The slides were air-dried and the bacteria were fixed by heat. The fixed cells were incubated with anti-His-DUF1542 antiserum or preimmune serum diluted 1∶500 in PBS for 2 h at 37°C in a humid chamber, washed 4 times with PBS, and then incubated with FITC-conjugated anti-rabbit F(ab′)_2_ fragment diluted 1∶500 for 1 h at 37°C in a humid chamber. The slides were washed twice with PBS and twice with double-distilled water. Then, the slides were dried, covered with fluorescent mounting medium (Dako, Hamburg, Germany), and viewed with a fluorescence microscope (Zeiss, Oberkochen, Germany).

### Nucleotide sequence accession number

The EMBL/GenBank/DDBJ accession number of the *sasC* DNA sequence of strain 4074 is FM202067.

## References

[1] Ziebuhr W (2001). *Staphylococcus aureus* and *Staphylococcus epidermidis*: emerging pathogens in nosocomial infections.. Contrib Microbiol.

[2] Lentino JR (2003). Prosthetic joint infections: bane of orthopedists, challenge for infectious disease specialists.. Clin Infect Dis.

[3] Mack D, Fischer W, Krokotsch A, Leopold K, Hartmann R (1996). The intercellular adhesin involved in biofilm accumulation of *Staphylococcus epidermidis* is a linear beta-1,6-linked glucosaminoglycan: purification and structural analysis.. J Bacteriol.

[4] Kogan G, Sadovskaya I, Chaignon P, Chokr A, Jabbouri S (2006). Biofilms of clinical strains of *Staphylococcus* that do not contain polysaccharide intercellular adhesin.. FEMS Microbiol Lett.

[5] Rice KC, Mann EE, Endres JL, Weiss EC, Cassat JE (2007). The *cidA* murein hydrolase regulator contributes to DNA release and biofilm development in *Staphylococcus aureus*.. Proc Natl Acad Sci U S A.

[6] Herrmann M, Lai QJ, Albrecht RM, Mosher DF, Proctor RA (1993). Adhesion of *Staphylococcus aureus* to surface-bound platelets: role of fibrinogen/fibrin and platelet integrins.. J Infect Dis.

[7] Biswas R, Voggu L, Simon UK, Hentschel P, Thumm G (2006). Activity of the major staphylococcal autolysin Atl.. FEMS Microbiol Lett.

[8] Heilmann C, Hussain M, Peters G, Götz F (1997). Evidence for autolysin-mediated primary attachment of *Staphylococcus epidermidis* to a polystyrene surface.. Mol Microbiol.

[9] Patti JM, Allen BL, McGavin MJ, Höök M (1994). MSCRAMM-mediated adherence of microorganisms to host tissues.. Annual Review of Microbiology.

[10] Clarke SR, Harris LG, Richards RG, Foster SJ (2002). Analysis of Ebh, a 1.1-megadalton cell wall-associated fibronectin-binding protein of *Staphylococcus aureus*.. Infect Immun.

[11] Flock JI, Froman G, Jonsson K, Guss B, Signas C (1987). Cloning and expression of the gene for a fibronectin-binding protein from *Staphylococcus aureus*.. Embo J.

[12] Jönsson K, Signas C, Müller HP, Lindberg M (1991). Two different genes encode fibronectin binding proteins in *Staphylococcus aureus*. The complete nucleotide sequence and characterization of the second gene.. Eur J Biochem.

[13] McDevitt D, Francois P, Vaudaux P, Foster TJ (1994). Molecular characterization of the clumping factor (fibrinogen receptor) of *Staphylococcus aureus*.. Mol Microbiol.

[14] Patti JM, Jonsson H, Guss B, Switalski LM, Wiberg K (1992). Molecular characterization and expression of a gene encoding a *Staphylococcus aureus* collagen adhesin.. J Biol Chem.

[15] Löfdahl S, Guss B, Uhlen M, Philipson L, Lindberg M (1983). Gene for staphylococcal protein A.. Proc Natl Acad Sci USA.

[16] Tung H, Guss B, Hellman U, Persson L, Rubin K (2000). A bone sialoprotein-binding protein from *Staphylococcus aureus*: a member of the staphylococcal Sdr family.. Biochem J.

[17] Heilmann C, Schweitzer O, Gerke C, Vanittanakom N, Mack D (1996). Molecular basis of intercellular adhesion in the biofilm-forming *Staphylococcus epidermidis*.. Mol Microbiol.

[18] Cramton SE, Gerke C, Schnell NF, Nichols WW, Götz F (1999). The intercellular adhesion (*ica*) locus is present in *Staphylococcus aureus* and is required for biofilm formation.. Infect Immun.

[19] Hussain M, Herrmann M, von Eiff C, Perdreau-Remington F, Peters G (1997). A 140-kilodalton extracellular protein is essential for the accumulation of *Staphylococcus epidermidis* strains on surfaces.. Infect Immun.

[20] Rohde H, Burdelski C, Bartscht K, Hussain M, Buck F (2005). Induction of *Staphylococcus epidermidis* biofilm formation via proteolytic processing of the accumulation-associated protein by staphylococcal and host proteases.. Mol Microbiol.

[21] Corrigan RM, Rigby D, Handley P, Foster TJ (2007). The role of *Staphylococcus aureus* surface protein SasG in adherence and biofilm formation.. Microbiology.

[22] Cucarella C, Solano C, Valle J, Amorena B, Lasa I (2001). Bap, a *Staphylococcus aureus* surface protein involved in biofilm formation.. J Bacteriol.

[23] Rohde H, Burandt EC, Siemssen N, Frommelt L, Burdelski C (2007). Polysaccharide intercellular adhesin or protein factors in biofilm accumulation of *Staphylococcus epidermidis* and *Staphylococcus aureus* isolated from prosthetic hip and knee joint infections.. Biomaterials.

[24] Bae T, Schneewind O (2003). The YSIRK-G/S motif of staphylococcal protein A and its role in efficiency of signal peptide processing.. J Bacteriol.

[25] Baba T, Takeuchi F, Kuroda M, Yuzawa H, Aoki K (2002). Genome and virulence determinants of high virulence community-acquired MRSA.. Lancet.

[26] Holden MT, Feil EJ, Lindsay JA, Peacock SJ, Day NP (2004). Complete genomes of two clinical *Staphylococcus aureus* strains: evidence for the rapid evolution of virulence and drug resistance.. Proc Natl Acad Sci U S A.

[27] Diep BA, Gill SR, Chang RF, Phan TH, Chen JH (2006). Complete genome sequence of USA300, an epidemic clone of community-acquired meticillin-resistant *Staphylococcus aureus*.. Lancet.

[28] Gill SR, Fouts DE, Archer GL, Mongodin EF, Deboy RT (2005). Insights on evolution of virulence and resistance from the complete genome analysis of an early methicillin-resistant *Staphylococcus aureus* strain and a biofilm-producing methicillin-resistant *Staphylococcus epidermidis* strain.. J Bacteriol.

[29] Baba T, Bae T, Schneewind O, Takeuchi F, Hiramatsu K (2008). Genome sequence of *Staphylococcus aureus* strain Newman and comparative analysis of staphylococcal genomes: polymorphism and evolution of two major pathogenicity islands.. J Bacteriol.

[30] Gillaspy AF, Worrell V, Orvis J, Roe BA, Dyer DW, Fischetti V, Novick R, Ferretti J, Portnoy D, Rood JI, (2006). The *Staphylococcus aureus* NCTC8325 genome.. Gram Positive Pathogens. 2nd ed.

[31] Kuroda M, Ohta T, Ushiyama I, Baba T, Yuzawa H (2001). Whole genome sequencing of meticillin-resistant *Staphylococcus aureus*.. Lancet.

[32] Wu SW, De Lencastre H (1999). Mrp–a new auxiliary gene essential for optimal expression of methicillin resistance in *Staphylococcus aureus*.. Microb Drug Resist.

[33] Komatsuzawa H, Ohta K, Sugai M, Fujiwara T, Glanzmann P (2000). Tn551-mediated insertional inactivation of the *fmtB* gene encoding a cell wall-associated protein abolishes methicillin resistance in *Staphylococcus aureus*.. J Antimicrob Chemother.

[34] Williams RJ, Henderson B, Sharp LJ, Nair SP (2002). Identification of a fibronectin-binding protein from *Staphylococcus epidermidis*.. Infect Immun.

[35] Horsburgh MJ, Aish JL, White IJ, Shaw L, Lithgow JK (2002). sigmaB modulates virulence determinant expression and stress resistance: characterization of a functional *rsbU* strain derived from *Staphylococcus aureus* 8325-4.. J Bacteriol.

[36] Mack D, Siemssen N, Laufs R (1992). Parallel induction by glucose of adherence and a polysaccharide antigen specific for plastic-adherent *Staphylococcus epidermidis*: evidence for functional relation to intercellular adhesion.. Infection and Immunity.

[37] Uhlen M, Guss B, Nilsson B, Gatenbeck S, Philipson L (1984). Complete sequence of the staphylococcal gene encoding protein A. A gene evolved through multiple duplications.. J Biol Chem.

[38] Fujigaki Y, Yousif Y, Morioka T, Batsford S, Vogt A (1998). Glomerular injury induced by cationic 70-kD staphylococcal protein; specific immune response is not involved in early phase in rats.. The Journal of Pathology.

[39] Gross M, Cramton SE, Götz F, Peschel A (2001). Key role of teichoic acid net charge in *Staphylococcus aureus* colonization of artificial surfaces.. Infect Immun.

[40] O'Neill E, Pozzi C, Houston P, Humphreys H, Robinson DA (2008). A novel *Staphylococcus aureus* biofilm phenotype mediated by the fibronectin-binding proteins, FnBPA and FnBPB.. J Bacteriol.

[41] Savolainen K, Paulin L, Westerlund-Wikstrom B, Foster TJ, Korhonen TK (2001). Expression of *pls*, a gene closely associated with the *mecA* gene of methicillin-resistant *Staphylococcus aureus*, prevents bacterial adhesion in vitro.. Infect Immun.

[42] Conrady DG, Brescia CC, Horii K, Weiss AA, Hassett DJ (2008). A zinc-dependent adhesion module is responsible for intercellular adhesion in staphylococcal biofilms.. Proc Natl Acad Sci U S A.

[43] Pascual A, Fleer A, Westerdaal NA, Verhoef J (1986). Modulation of adherence of coagulase-negative staphylococci to Teflon catheters in vitro.. Eur J Clin Microbiol.

[44] Heilmann C, Gerke C, Perdreau-Remington F, Götz F (1996). Characterization of Tn*917* insertion mutants of *Staphylococcus epidermidis* affected in biofilm formation.. Infect Immun.

[45] Clarke SR, Brummell KJ, Horsburgh MJ, McDowell PW, Mohamad SA (2006). Identification of in vivo-expressed antigens of *Staphylococcus aureus* and their use in vaccinations for protection against nasal carriage.. J Infect Dis.

[46] Yarwood JM, Bartels DJ, Volper EM, Greenberg EP (2004). Quorum sensing in *Staphylococcus aureus* biofilms.. J Bacteriol.

[47] Vuong C, Saenz HL, Götz F, Otto M (2000). Impact of the agr quorum-sensing system on adherence to polystyrene in *Staphylococcus aureus*.. J Infect Dis.

[48] Pratten J, Foster SJ, Chan PF, Wilson M, Nair SP (2001). *Staphylococcus aureus* accessory regulators: expression within biofilms and effect on adhesion.. Microbes Infect.

[49] Beenken KE, Blevins JS, Smeltzer MS (2003). Mutation of *sarA* in *Staphylococcus aureus* limits biofilm formation.. Infect Immun.

[50] Bateman BT, Donegan NP, Jarry TM, Palma M, Cheung AL (2001). Evaluation of a tetracycline-inducible promoter in *Staphylococcus aureus* in vitro and in vivo and its application in demonstrating the role of *sigB* in microcolony formation.. Infect Immun.

[51] Lim Y, Jana M, Luong TT, Lee CY (2004). Control of glucose- and NaCl-induced biofilm formation by *rbf* in *Staphylococcus aureus*.. J Bacteriol.

[52] Jefferson KK, Pier DB, Goldmann DA, Pier GB (2004). The teicoplanin-associated locus regulator (TcaR) and the intercellular adhesin locus regulator (IcaR) are transcriptional inhibitors of the *ica* locus in *Staphylococcus aureus*.. J Bacteriol.

[53] Fournier B, Hooper DC (2000). A new two-component regulatory system involved in adhesion, autolysis, and extracellular proteolytic activity of *Staphylococcus aureus*.. J Bacteriol.

[54] Tsang LH, Cassat JE, Shaw LN, Beenken KE, Smeltzer MS (2008). Factors contributing to the biofilm-deficient phenotype of *Staphylococcus aureus sarA* mutants.. PLoS ONE.

[55] Heilmann C, Herrmann M, Kehrel BE, Peters G (2002). Platelet-binding domains in 2 fibrinogen-binding proteins of *Staphylococcus aureus* identified by phage display.. J Infect Dis.

[56] Götz F, Kreutz B, Schleifer KH (1983). Protoplast transformation of *Staphylococcus carnosus* by plasmid DNA.. Mol Gen Genet.

[57] Horsburgh SM (2002). Identification of novel regulators of virulence determinant production in *Staphylococcus aureus*..

[58] Wieland K-P, Wieland B, Götz F (1995). A promotor-screening plasmid and xylose-inducible, glucose-repressible expression vectors for *Staphylococcus carnosus*.. Gene.

[59] Sambrook J, Fritsch EF, Maniatis T (1989). Molecular Cloning: A Laboratory Manual.. Cold Spring Harbor.

[60] Götz F, Schumacher B (1987). Improvements of protoplast transformation in *Staphylococcus carnosus*.. FEMS Microbiol Lett.

[61] Altschul SF, Madden TL, Schaffer AA, Zhang J, Zhang Z (1997). Gapped BLAST and PSI-BLAST: a new generation of protein database search programs.. Nucleic Acids Res.

[62] Pearson WR, Lipman DJ (1988). Improved tools for biological sequence comparison.. Proc Natl Acad Sci U S A.

[63] Finn RD, Mistry J, Schuster-Bockler B, Griffiths-Jones S, Hollich V (2006). Pfam: clans, web tools and services.. Nucleic Acids Res.

[64] Lahav J, Jurk K, Hess O, Barnes MJ, Farndale RW (2002). Sustained integrin ligation involves extracellular free sulfhydryls and enzymatically catalyzed disulfide exchange.. Blood.

[65] Kehrel B, Kronenberg A, Schwippert B, Niesing-Bresch D, Niehues U (1991). Thrombospondin binds normally to glycoprotein IIIb deficient platelets.. Biochem Biophys Res Commun.

[66] Hartleib J, Kohler N, Dickinson RB, Chhatwal GS, Sixma JJ (2000). Protein A is the von Willebrand factor binding protein on *Staphylococcus aureus*.. Blood.

[67] Dormann D, Clemetson KJ, Kehrel BE (2000). The GPIb thrombin-binding site is essential for thrombin-induced platelet procoagulant activity.. Blood.

[68] Niemann S, Spehr N, Van Aken H, Morgenstern E, Peters G (2004). Soluble fibrin is the main mediator of *Staphylococcus aureus* adhesion to platelets.. Circulation.

